# Supplementary material for: Concurrent TP53 and CDKN2A Gene Aberrations in Newly Diagnosed Mantle Cell Lymphoma Correlate with Chemoresistance and Call for Innovative Upfront Therapy
Source: Cancers (Basel). 2020 Jul 31;12(8):2120. doi: 10.3390/cancers12082120 (PMC7466084; doi:10.3390/cancers12082120)
Supplement: Supplementary file 1 [file cancers-12-02120-s001.pdf]

Supplementary Materials: Concurrent TP53 and CDKN2A Gene Aberrations in Patients with Newly Diagnosed Mantle Cell Lymphoma Correlate with Chemoresistance and Call for Innovative Upfront Therapy

Supplementary Methods:

Fluorescence *in Situ* Hybridization (FISH)

For conventional cytogenetic analyses, unstimulated bone marrow cells were cultivated for 24 hours in RPMI 1640 medium with 10% fetal calf serum. Chromosomal preparations were done according to standard techniques using colcemide, hypotonic treatment, fixation in methanol-acetic acid, and G-banding with Wright stain. Complex structural rearrangements were analyzed by multicolor FISH (mFISH) with the 24XCyte color kit (MetaSystems, Altlussheim, Germany). Chromosomal aberrations were described according to ISCN nomenclature (2016). Complex karyotypes were defined as containing numerical and/or structural changes involving three or more chromosomes and/or structural rearrangements involving three or more breaks in a single clone. Interphase FISH analyses were performed with commercially available DNA probes Vysis IGH/CCND1 XT DF, Vysis LSI MYC BA, Vysis LSI BCL2 BA; Vysis LSI 13q14 (RB1) SO/LSI 13q34 SG, Vysis LSI CDKN2A/CEP 9 (Abbott Molecular, Des Plaines, IL, USA), XL ATM/TP53 (MetaSystems, Altlussheim, Germany), and SPEC CDK4/CEN 12 DC (ZytoVision GmbH, Bremerhaven, Germany) according to the manufacturers' recommendations. At least 200 interphase nuclei were analyzed by two independent observers. In patients with ≥ 5 % but < 10 % infiltration of the bone marrow with lymphoma cells, 400 interphase nuclei were analyzed to reach the 5% threshold of the detection of the analyzed gene aberration. The cut-off level for positive values were determined on samples obtained from 10 cytogenetically normal persons and were found to be 5% (mean ± 3SD) for losses (deletions, monosomies), and 2.5% (mean ± 3SD) for translocations and gains (trisomies, duplications/amplifications).

Supplementary Results:

Table S1. Univariate analysis: correlation of analyzed gene aberrations and selected clinical and laboratory parameters with survival.

A. Event-free survival

Gene	HR	95% CI	*p*
TP53	2.2	1.5–4.0	<0.001
CDKN2A	3.0	2.4–6.8	<0.001
RB1	2.0	1.3–3.7	0.003
MYC	1.9	1.3–3.8	0.004
CDK4	2.3	1.5–7.5	0.005
BCL2	1.9	1.2–4.0	0.016
ATM	1.3	0.8–2.2	0.303

B. Overall survival

Gene	HR	95% CI	*p*
TP53	2.3	1.4–4.3	0.002
CDKN2A	3.2	2.4–8.1	<0.001
RB1	2.2	1.4–4.6	0.003
MYC	1.7	1.0–3.5	0.068
CDK4	3.0	2.2–18.2	0.001
BCL2	2.4	2.3–10.9	<0.001
ATM	1.2	0.7–2.3	0.504

C. Event-free survival

Gene	HR	95% CI	*p*
**Male sex**	1.5	0.9–2.3	0.144
**Ki-67**	2.0	1.2–3.8	0.011
**B-symptoms**	2.7	1.9–5.1	<0.001
**Nodal involvement**	3.4	1.1–4.3	0.026
**EH involvement**	1.5	1.0–2.5	0.062
**Splenomegaly**	1.8	1.0–2.8	0.047
**Bulky disease**	1.9	1.3–3.4	0.005
**Complex karyotype**	2.7	1.4–14.7	0.014

D. Overall survival

Gene	HR	95% CI	*p*
**Male sex**	2.1	1.1–3.3	0.032
**Ki-67**	2.2	1.1–4.7	0.026
**B-symptoms**	2.8	1.8–5.5	<0.001
**Nodal involvement**	1.9	0.7–4.0	0.255
**EH involvement**	1.5	0.9–2.7	0.149
**Splenomegaly**	1.2	0.6–2.2	0.624
**Bulky disease**	1.5	0.8–2.7	0.185
**Complex karyotype**	3.9	2.9–58.7	0.001

Legend: EH = extrahematological involvement, i.e., extranodal involvement besides bone marrow involvement; statistically significant results are highlighted in gray.

Table S2. Distribution of the analyzed gene aberrations.

Legend: Numbers represent type of aberration: 0 = not available / not able to analyze, 1 = normal finding, 2 = monoallelic deletion, 3 = bialellic deletion, 4 = monosomy, 5 = nullisomy, 6 = amplification, 7 = gain, 8 = trisomy, 9 = tetrasomy, 10 = *MYC* rearrangement, 11 = t(11;14), 12 = *CCND1* rearrangement, 13 = complex conventional caryotype, 14 = *TP53* mutation, Y = yes, N = no

Patient ID	t(11;14)(q13;q32)	t(11;14) [%]	Complex conventional karyotype	*CDK4*	*CDK4* [%]	*RB1*	*RB1* [%]	*BCL2*	*BCL2* [%]	*ATM*	*ATM* [%]	*MYC*	*MYC* [%]	*CDKN2A*	*CDKN2A* [%]	*TP53*	*TP53* [%]	*TP53* MUTATION	VAF [%]	*TP53* ABERRATION
1	11	84	1	1		1		1		2	80	1		2 and 3	ion - 8, bial	1		1		N
2	11	11	1	1		4	15	1		1		1		1		1		0		N
3	11	20	1	1		1		1		1		1		1		1		1		N
4	11	0	1	1		1		1		1		1		1		2	12	1		Y
5	11	29	1	1		1		1		1		1		2	28	2	24	14	16	Y
6	11	80	0	1		2 and 4	my - 13, del	1		1		1		4 and 4+2	osomy + m	2	87	14	17	Y
7	11	43	1	1		4	70	1		2	68	1		2	66	2	75	14	78	Y
8	11	68	0	1		2	30	1		1		7	63	1		2	56	14	66	Y
9	11	39	0	1		1		7	12	2	16	7	16	1		1		1		N
10	11	91	1	1		4	90	1		1		1		4	90	1		0		N
11	11	12	1	1		1		1		2	14	7	15	3	18	1		1		N
12	11	51	1	1		1		1		1		1		1		1		1		N
13	11	27	13	8	30	2	30	1		1		10	24	3	24	1		1		N
14	11	77	1	1		1		1		1		1		1		1		1		N
15	11	7	1	1		1		1		1		1		1		2	6	14	5	Y
16	11	7	1	1		1		1		1		1		4	7	1		1		N
17	11	0	1	1		1		1		1		1		1		2	28	14	10	Y
18	11	94	13	1		1		1		2	20	10	12	2	12	2	92	14	85	Y
19	11	57	1	1		1		7	55	1		1		1		2	53	14	33	Y
20	11	22	0	1		1		1		1		1		1		1		1		N
21	11	30	1	1		4	30	1		1		1		1		1		1		N
22	11	7	1	1		1		1		2	8	1		1		1		1		N
23	11	87	0	1		2	8	1		1		1		1		2	8	14	3	Y
24	11	21	1	1		1		1		1		1		1		1		1		N
25	11	33	1	1		4	40	1		2	30	7	18	2	33	1		1		N
26	11	51	13	9	50	4	50	7	50	2	45	1		4	51	1		14	65	Y
27	11	11	1	1		1		7	10	1		7	10	1		1		0		N
28	11	77	0	1		1		1		1		1		1		2	6	14	29+3	Y
29	11	90	0	1		1		1		1		1		1		1		14	26+13+7+5+	Y
30	11	24	1	1		1		1		2	31	1		1		1		1		N
31	12	eak, t(11;14	1	1		1		1		1		1		1		1		1		N
32	11	37	13	1		1		7	3	1		1		3	37	1		1		N
33	11	34	1	1		1		1		1		1		1		1		1		N
34	11	60	1	7	60	4	60	7	15	2	51	1		2	45	1		1		N
35	11	79	1	1		1		1		1		1		2	10	2	85	14	71	Y
36	11	71	0	1		4	25	7	15	1		7	20	1		2	79	0		Y
37	11	31	0	1		1		1		1		1		1		1		1		N
38	11	54	1	1		1		7	50	2	51	6	50	1		2	51	14	44	Y
39	11	35	1	1		1		1		1		1		1		1		1		N
40	11	81	1	1		1		1		1		7	45	1		1		1		N
41	11	16	1	1		4	24	7	24	1		1		4	19	1		14	26	Y
42	11	84	0	1		1		1		1		1		1		1		14	80	Y
43	11	14	1	1		1		1		2	17	1		1		1		1		N
44	12	73	0	9	75	1		7	CL2/+2 BCL2	2	13	1		2	39	2	77	14	28	Y
45	11	73	1	1		2	na	7	74	1		1		2	24	1		1		N
46	11	90	0	1		1		1		1		1		1		1		0		N
47	11	78	1	1		4	80	1		2	79	1		4 and 2	ny - 71, dele	1		14	35	Y
48	11	11	1	1		4	15	1		1		1		4	7	1		14	31	Y
49	11	21	1	1		1		1		1		1		1		1		1		N
50	11	32	1	1		2	35	1		1		7	31	2	41	2	35	14	18	Y
51	11	40	1	1		1		7	14	1		10 and 7	ement - 6,	4	63	2	60	0		Y
52	11	90	1	1		4	20	1		2	88	1		1		1		1		N
53	11	92	0	9	90	4	11	6	90	1		7	95	5	85	1		0		N
54	11	20	1	1		1		1		1		1		1		1		14	18+8	Y
55	11	41	1	1		1		1		1		1		1		2	35	14	34	Y
56	11	28	1	1		1		1		2	25	1		1		1		14	3	Y
57	11	13	0	1		2	16	1		1		1		1		1		0		N
58	11	42	1	1		1		1		1		1		1		1		1		N
59	11	19	1	1		1		1		1		1		1		1		1		N
60	11	88	0	1		1		1		1		1		1		2	93	14	82	Y
61	11	72	1	7	40	1		1		1		1		1		1		1		N
62	11	7	1	1		1		1		1		1		1		1		1		N
63	11	6	1	1		1		1		2	19	1		1		1		1		N
64	11	50	1	1		2 and 4	14 - 40, mo	1		2	70	1		2 and 3	on - 35, bia	1		1		N
65	11	5	1	1		1		1		1		1		1		1		1		N
66	11	15	1	1		2	15	1		2	8	1		2	7	1		1		N
67	11	39	1	7	8	1	0	7	8	2	33	7	37	1		1		1		N
68	11	15	1	1		1		1		1		1		1		1		1		N
69	11	5	1	1		1		7	2	1		1		1		1		1		N
70	11	80	1	1		2	60	1		1		1		4	75	2	80	14	48	Y
71	11	87	0	1		4	90	1		1		1		2	13	1		0		N
72	11	75	13	1		1		6	60	1		1		2	75	1		1		N
73	11	41	1	1		1		1		1		1		1		2	59	14	22+10	Y
74	11	10	1	1		1		7	10	1		1		1		1		1		N
75	11	11	1	1		1		7	7	1		1		1		1		1		N
76	11	10	1	8	10	1		1		1		1		1		1		1		N
77	11	54	1	1		1		1		1		1		1		2	61	1		Y
78	11	80	0	1		1		1		1		7	50	1		1		1		N
79	11	25	0	1		1		1		2	25	1		1		1		0		N
80	11	15	0	1		1		1		1		1		1		1		1		N
81	11	53	1	1		1		1		1		1		1		1		1		N
82	11	40	1	1		1		1		1		7	22	1		1		1		N
83	11	47	1	1		1		1		2	47	1		1		1		1		N
84	12	56	1	1		1		1		2	85	7	29	1		1		1		N
85	11	50	1	1		1		1		1		7	45	1		1		14	27	Y
86	11	5	1	1		4	5	1		1		1		1		1		1		N
87	11	94	0	8	11	1		1		1		7	8	2	6	2	93	14	88	Y
88	11	50	1	1		2	50	1		1		1		1		1		1		N
89	11	11	1	1		1		1		1		1		1		1		1		N
90	11	13	1	1		2	20	1		1		1		4	21	2	23	14	19	Y
91	11	60	0	1		1		1		2	48	10	2	1		1		1		N
92	11	9	13	1		2 and 4	10, deletio	1		1		1		4	10	2	11	1		Y
93	11	35	1	1		4	36	1		1		7	32	4	29	2	33	14	20	Y
94	11	88	1	1		1		6	80	2	89	1		2 and 3	ion - 79, bia	1		1		N
95	11	61	0	1		1		1		1		7	60	1		2	66	14	18	Y
96	11	22	1	1		1		1		1		1		1		1		1		N
97	11	10	1	1		1		1		1		1		1		1		1		N
98	11	16	1	1		4	20	1		1		7	24	1		1		1		N
99	11	69	1	1		1		1		1		1		2	26	2	66	0		Y
100	11	49	1	1		1		1		1		7	50	1		2	25	14	33	Y
101	11	13	1	7	13	1		7	8	2	8	1		1		1		1		N
102	11	60	1	1		1		1		2	25	1		1		1		1		N
103	11	72	0	1		1		1		1		1		1		2	70	14	45+4	Y
104	11	60	1	7	8	4	60	7	60	1		7	8	4	57	1		14	78	Y
105	11	72	1	1		1		1		1		7	7	1		1		14	8	Y
106	11	53	0	8	20	2	9	7	97	2	93	1		2	97	2	100	14	2	Y
107	11	34	1	1		1		1		2	21	1		1		1		1		N
108	11	60	1	1		1		1		2	50	1		1		2	50	0		Y
109	11	85	0	1		1		1		2	85	7	85	3	85	2	85	1		Y
110	11	84	0	1		2	7	1		1		7	6	1		2	27	1		Y
111	11	72	0	1		4	72	1		1		7	15	2	30	2	72	14	55	Y
112	11	21	1	8	30	1		1		1		1		1		1		1		N
113	11	58	1	1		1		1		1		1		1		1		1		N
114	11	9	0	1		1		1		1		1		1		1		1		N
115	11	64	0	1		1		1		1		1		1		1		0		N
116	11	16	1	1		1		1		1		1		1		2	11	14	11	Y
117	11	61	13	and 8 and 9		1		7	43	1		7	48	1		1		14	47	Y
118	11	38	1	1		2	40	7	40	2	42	7	40	4	40	1		1		N
119	11	95	1	1		1		1		1		1		1		2	96	14	84	Y
120	11	69	1	1		1		1		1		1		1		1		1		N
121	11	53	1	1		1		1		2	6	1		4	5	2	82	14	13	Y
122	11	63	1	1		4	56	1		2	73	1		1		1		1		N
123	11	43	1	1		1		1		2	86	1		1		1		1		N
124	11	10	1	1		2	7	1		1		7	11	2	16	1		1		N
125	11	71	1	8	63	4	58	1		1		7	5	4	18	2	56	14	33	Y
126	11	32	1	1		1		1		1		1		1		2	40	14	15	Y

Table S3. *TP53* mutation types and positions.

Patient ID	Position (hg38) with nucleotide change	cDNA	Protein	*TP53* mutation type	COSMIC	VAF (%)
5	g.[7675055_7675078delTCGCTATCTGAGCAGCGCTCATGG;7675081G>T]	c.[531C>A;534_557delCCATGACGGCTGCTCAGATAGCGA]	p.H178_D18	inframe		16
6	g.7675161G>A	c.451C>T	p.P151S	missense		17
7	g.[7675085C>A;7675086A>G]	c.[526T>C;527G>T]	p.C176L	missense		78
8	g.7673767C>T	c.853G>A	p.E285K	missense		66
15	g.7674250C>A	c.713G>T	p.C238F	missense		5
17	g.7675236A>C	c.376T>G	p.Y126D	missense/splice region		10
18	g.7674945G>A	c.586C>T	p.R196*	nonsense		85
19	g.7673776G>C	c.844C>G	p.R282G	missense		33
23	g.7675209delA	c.403delT	p.C135fs	frameshift		3
26	g.7674945G>A	c.586C>T	p.R196*	nonsense		65
28	g.7673803G>A	c.817C>T	p.R273C	missense		6
	g.7674229C>T	c.734G>A	p.G245D	missense		29
	g.7674917T>C	c.614A>G	p.Y205C	missense		3
29	g.7673806C>T	c.814G>A	p.V272M	missense		13
	g.7674220C>G	c.743G>C	p.R248P	missense		7
	g.7674220C>T	c.743G>A	p.R248Q	missense		5
	g.7675094A>G	c.518T>C	p.V173A	missense		26
	g.7673799A>T	c.821T>A	p.V274D	missense		3
35	g.7673802C>G	c.818G>C	p.R273P	missense		71
38	g.7674885C>T	c.646G>A	p.V216M	missense		44
41	g.7674256T>C	c.707A>G	p.Y236C	missense		26
42	g.7675088C>T	c.524G>A	p.R175H	missense		80
44	g.7673704G>A	c.916C>T	p.R306*	nonsense		28
47	g.7675076T>C	c.536A>G	p.H179R	missense		35
48	g.7670681delT	c.1028delA	p.E343fs	frameshift	COSM6907052	31
50	g.7673806C>T	c.814G>A	p.V272M	missense		18
54	g.7674263A>G	c.700T>C	p.Y234H	missense		8
	g.7674885C>T	c.646G>A	p.V216M	missense		9
55	g.7674263A>T	c.700T>A	p.Y234N	missense		34
56	g.7673805A>T	c.815T>A	p.V272E	missense		3
60	g.7674220C>T	c.743G>A	p.R248Q	missense		82
70	g.7673782T>C	c.838A>G	p.R280G	missense		48
73	g.7670685G>A	c.1024C>T	p.R342*	nonsense		10
	g.7673610T>C	c.920-2A>G	-	splice region		22
85	g.7673728C>A	c.892G>T	p.E298*	nonsense	COSM10710	27
87	g.7674238C>G	c.725G>C	p.C242S	missense	COSM11133	88
90	g.7674962delG	c.569delC	p.P190fs	frameshift		19
93	g.7675088C>T	c.524G>A	p.R175H	missense		20
95	g.7673778T>A	c.842A>T	p.D281V	missense		48
100	g.7669692T>A	c.1101-2A>T	-	splice region	COSM45409	33
103	g.7674220C>A	c.743G>T	p.R248L	missense		45
	g.7670703C>A	c.1006G>T	p.E336*	nonsense	COSM11291	4
104	g.7673534C>T	c.993+1G>A	-	splice region		78
105	g.7674890T>C	c.641A>G	p.H214R	missense		8
106	g.7674225C>A	c.738G>T	p.M246I	missense		2
111	g.7674953T>A	c.578A>T	p.H193L	missense		55
116	g.7674201_7674209delGATGGTGAG	c.754_762delCTCACCATC	p.L252_I254	inframe	COSM45333	11
117	g.7673704G>A	c.916C>T	p.R306*	nonsense		47
119	g.7674872T>C	c.659A>G	p.Y220C	missense		84
121	g.7674858C>T	c.672+1G>A	-	splice region		13
125	g.7674252C>T	c.711G>A	p.M237I	missense		33
126	g.7674887C>A	c.644G>T	p.S215I	missense		15

Legend: VAF = variant allele frequency

Table S4. Univariate analysis of *TP53* mutation and *TP53* deletion.

A. Event-free survival

Gene	HR	95% CI	*p*
***TP53* del**	2.3	1.2–32.8	0.04
***TP53* mut**	3	1.9–15.1	0.002
***TP53* del+mut**	2.3	1.5–5.3	0.002

B. Overall survival.

Column Title	HR	95% CI	*p*
***TP53* del**	3.8	1.5–105.6	0.021
***TP53* mut**	3.7	2.6–26.9	<0.001
***TP53* del+mut**	2.1	1.1–5.1	0.024

Table S5. Baseline characteristics and response to therapy of the patients with concurrent aberration of TP53 and CDKN2A (compared to remaining patients).

Gene	*TP53*$^{del/mut}$ + *CDKN2A*^{del}		The Remaining Pts with Bone Marrow Involvement ≥ 5%	
**Gene**	*N*	%	*N*	%
**All patients**	24	19	102	81
**M**	19	79	69	68
**F**	5	21	33	32
**Age (median; years)**	70		67	
**Age (range; years)**	46–79		29–82	
**<65 years**	8	33	39	38
**≥65 years**	16	67	64	63
**Ki-67 ≥ 30%***	11	85	25	40
**MIPI 1**	1	4	18	18
**MIPI 2**	4	17	25	25
**MIPI 3**	19	79	59	58
**B-symptoms**	17	71	35	34
**Nodal involvement**	21	88	87	85
**Splenomegaly**	19	79	70	69
**Extra-hematological involvement**	11	46	39	38
**Bulky disease (≥5 cm)**	11	46	34	33
**CNS involvement****	8	33	9	9
**Intensified therapy**	8	33	29	28
**R-CHOP-like therapy**	10	42	61	60
**Palliative therapy**	5	21	3	3
**Watch and wait**	0	0	7	7
**Died before initiation of therapy**	1	4	2	2
**Died during induction*****	4	17	5	5
**ORR (CR/PR)**	9	38	81	79
**CR**	4	17	57	56
**PR**	5	21	24	24
**SD**	3	13	1	1
**PD**	7	29	8	8
**Event**	23	96	55	54
**Relapse**	17	71	36	35
**Death****	19	79	36	35

Legend: M = male; F = female; MIPI = MCL international prognostic index; BM = bone marrow; CNS = central nervous system; ORR = overall response rate; CR = complete remission; PR = partial remission; SD = stable disease; PD = progressive disease; response was assessed by international workshop criteria published by Cheson et al. in 1999(7); * of the analyzed patients, ** anytime from diagnosis until database lock, *** after initiation of therapy, before restaging; differences >20% between cohorts are highlighted in gray

Figure S1. Survival parameters in the *TP53* mutation and *TP53* deletion cohorts.

A. Event-free survival

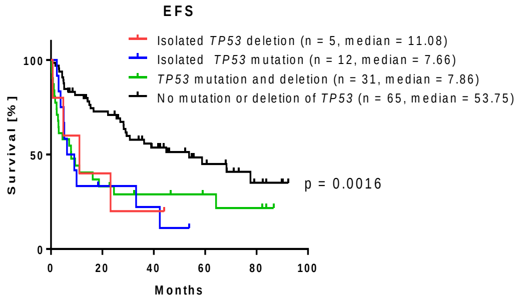

B. Overall survival

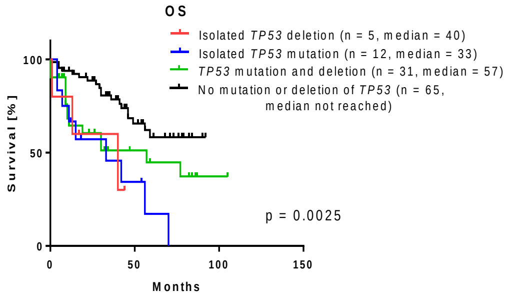

Legend: Subcohort "Isolated *TP53* deletion" includes all patients with deletion of *TP53*, but without mutation of *TP53*, while in subcohort "Isolated *TP53* mutation" are patients with detected mutation of *TP53*, but without deletion. Subgroup "*TP53* mutation and deletion" includes 31 patients with mutation and deletion of *TP53*. Only 113 patients investigated by both FISH and NGS were included in this analysis. EFS = event-free survival, OS = overall survival

Figure S2. Random Forest analysis of analyzed aberrations.

A:

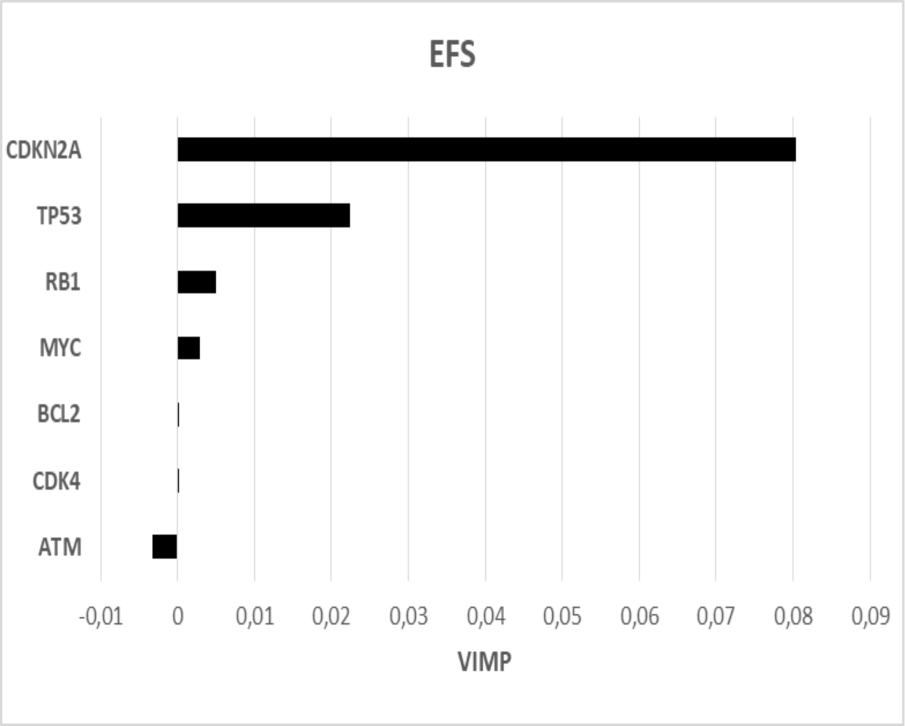

B:

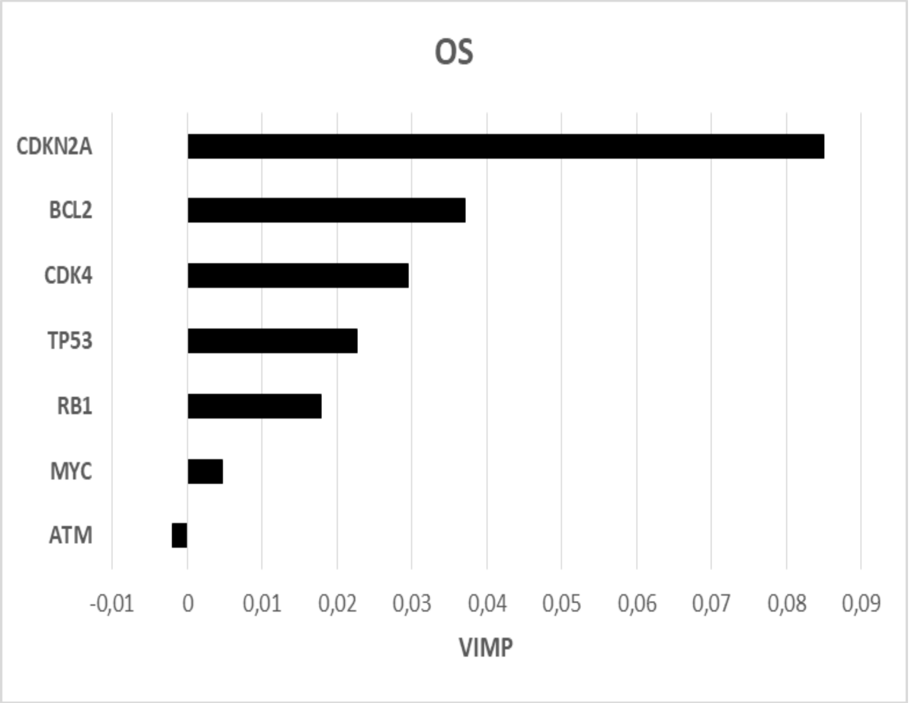

C:

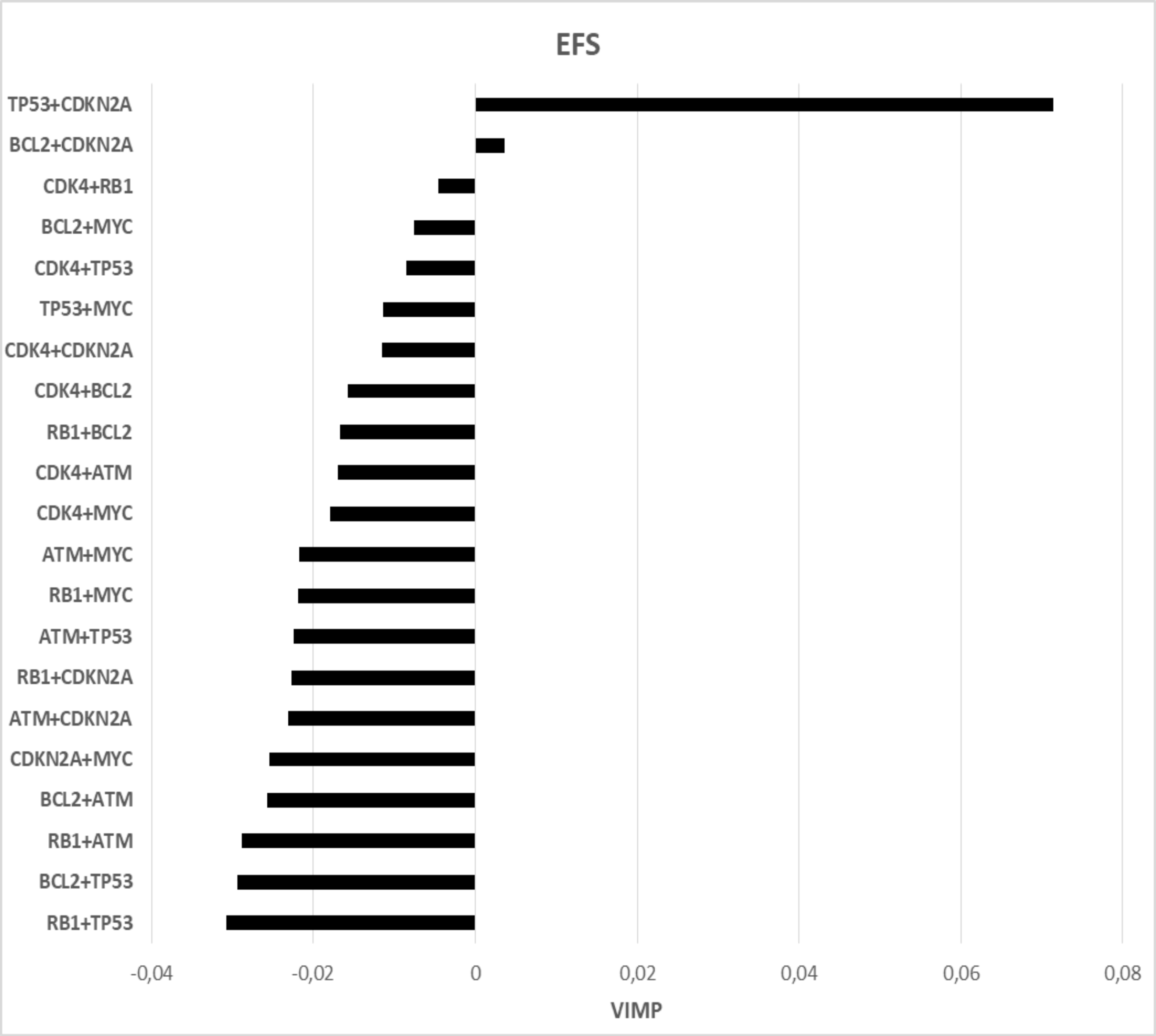
EFS
TP53+CDKN2A
BCL2+CDKN2A
CDK4+RB1
BCL2+MYC
CDK4+TP53
TP53+MYC
CDK4+CDKN2A
CDK4+BCL2
RB1+BCL2
CDK4+ATM
CDK4+MYC
ATM+MYC
RB1+MYC
ATM+TP53
RB1+CDKN2A
ATM+CDKN2A
CDKN2A+MYC
BCL2+ATM
RB1+ATM
BCL2+TP53
RB1+TP53
-0,04
-0,02
0
0,02
0,04
0,06
0,08
VIMP

D:

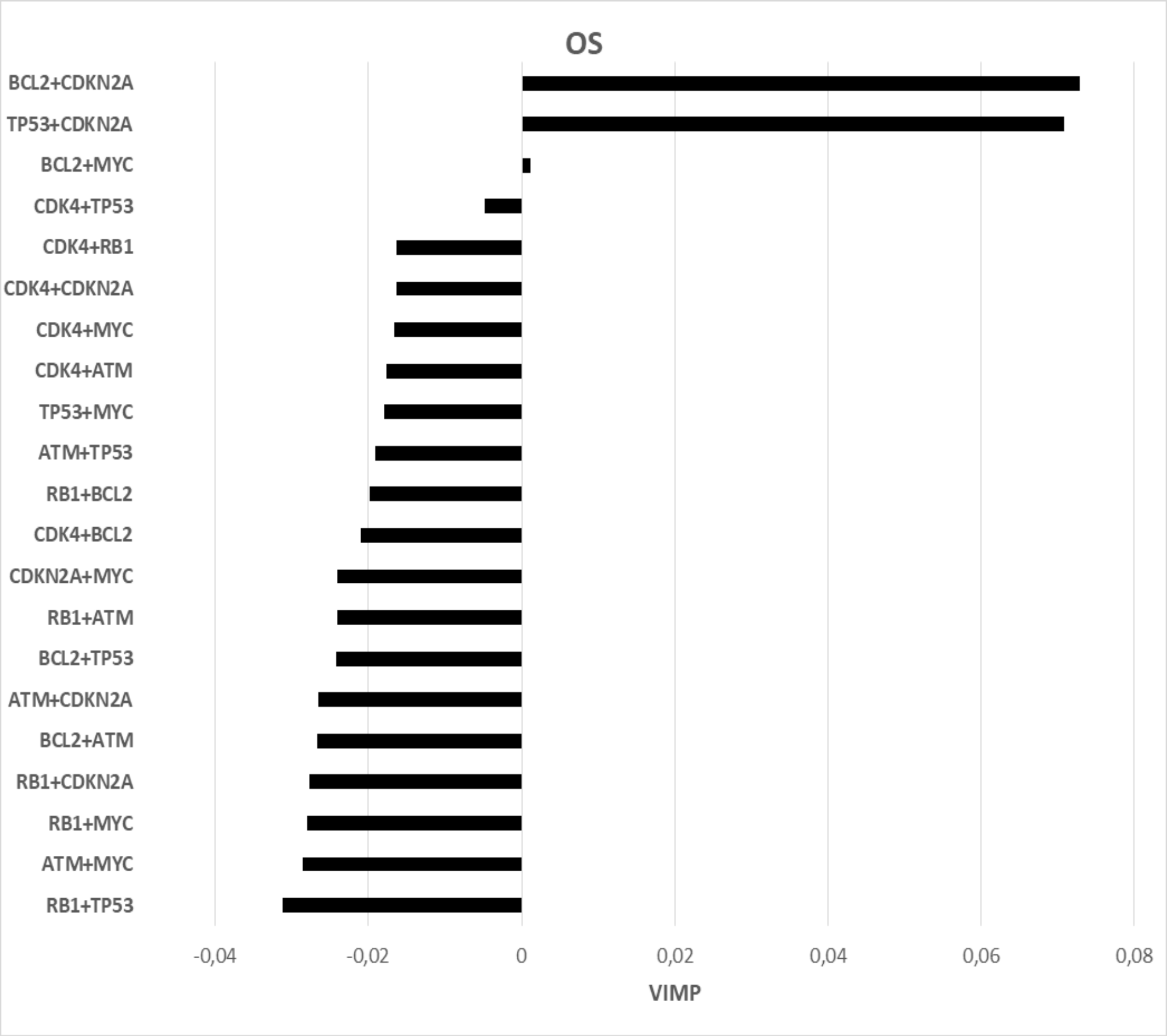

Legend: Positive VIMP values indicate that the variable increases the prediction accuracy of random forest analysis, whereas negative or near-zero values have no effect on survival prediction. EFS = event-free survival; OS = overall survival.
